# Arsenic Trioxide Prevents Osteosarcoma Growth by Inhibition of GLI Transcription via DNA Damage Accumulation

**DOI:** 10.1371/journal.pone.0069466

**Published:** 2013-07-08

**Authors:** Shunsuke Nakamura, Satoshi Nagano, Hiroko Nagao, Yasuhiro Ishidou, Masahiro Yokouchi, Masahiko Abematsu, Takuya Yamamoto, Setsuro Komiya, Takao Setoguchi

**Affiliations:** 1 Department of Orthopaedic Surgery, Graduate School of Medical and Dental Sciences, Kagoshima University, Kagoshima, Japan; 2 Department of Medical Joint Materials, Graduate School of Medical and Dental Sciences, Kagoshima University, Kagoshima, Japan; 3 The Near-Future Locomotor Organ Medicine Creation Course (Kusunoki Kai),Graduate School of Medical and Dental Sciences, Kagoshima University, Kagoshima, Japan; Peking University Health Science Center, China

## Abstract

The Hedgehog pathway is activated in various types of malignancies. We previously reported that inhibition of SMO or GLI prevents osteosarcoma growth in vitro and in vivo. Recently, it has been reported that arsenic trioxide (ATO) inhibits cancer growth by blocking GLI transcription. In this study, we analyzed the function of ATO in the pathogenesis of osteosarcoma. Real-time PCR showed that ATO decreased the expression of Hedgehog target genes, including *PTCH1*, *GLI1*, and *GLI2*, in human osteosarcoma cell lines. WST-1 assay and colony formation assay revealed that ATO prevented osteosarcoma growth. These findings show that ATO prevents GLI transcription and osteosarcoma growth in vitro. Flow cytometric analysis showed that ATO promoted apoptotic cell death. Comet assay showed that ATO treatment increased accumulation of DNA damage. Western blot analysis showed that ATO treatment increased the expression of γH2AX, cleaved PARP, and cleaved caspase-3. In addition, ATO treatment decreased the expression of Bcl-2 and Bcl-xL. These findings suggest that ATO treatment promoted apoptotic cell death caused by accumulation of DNA damage. In contrast, Sonic Hedgehog treatment decreased the expression of γH2AX induced by cisplatin treatment. ATO re-induced the accumulation of DNA damage attenuated by Sonic Hedgehog treatment. These findings suggest that ATO inhibits the activation of Hedgehog signaling and promotes apoptotic cell death in osteosarcoma cells by accumulation of DNA damage. Finally, examination of mouse xenograft models showed that ATO administration prevented the growth of osteosarcoma in nude mice. Because ATO is an FDA-approved drug for treatment of leukemia, our findings suggest that ATO is a new therapeutic option for treatment of patients with osteosarcoma.

## Introduction

Osteosarcoma is the most common malignant bone tumor in children and adolescents [1,2]. Osteosarcoma is a highly aggressive neoplasm that is resistant to current therapeutic approaches, including radiation, chemotherapy, and surgical treatment. The survival rate of patients treated with neoadjuvant chemotherapy and local control therapy is 60–80% [3]. The predicted outcome is poor in patients with lung metastasis at first diagnosis, with long-term survival rates ranging between 10% and 40% [4]. Therefore, more effective treatments and more personalized therapies (i.e., treatments targeting a specific signaling pathway or gene) are essential for patients with osteosarcoma.

The Hedgehog pathway is involved in various aspects of development. The Hedgehog pathway is activated via the PATCHED (PTCH1) and SMOOTHENED (SMO) Hedgehog receptors. Activation of SMO promotes the activation of GLI family transcription factors (GLI1, GLI2, and GLI3) to regulate the transcription of target genes [5–7]. Aberrant activation of the Hedgehog pathway is associated with malignant tumors (reviewed in ref [8].). We have previously reported that aberrant activation of the Hedgehog pathway is involved in the pathoetiology of osteosarcoma. Inhibition of the Hedgehog pathway by knockdown of SMO or GLI2 prevents osteosarcoma growth in vitro and in vivo [9,10]. Although several SMO inhibitors have been developed, they have several limitations, including constitutive activation of SMO, spontaneous mutation of SMO that impairs its binding to the drug, and constitutive activation downstream of SMO [11–21]. Arsenic trioxide (ATO) is an FDA-approved drug used for the treatment of patients with acute promyelocytic leukemia (APL) who show relapse after first-line chemotherapy (reviewed in 22. ATO promotes complete remission without myelosuppression and causes few adverse reactions. Recently, it has been reported that ATO prevents human cancer cell growth by inhibiting activation of the Hedgehog pathway [23–25]. In the present study, we examined the effect of ATO treatment on GLI transcription and osteosarcoma growth in vitro and in vivo. Our findings show that ATO inhibits Hedgehog pathway signaling and prevents human osteosarcoma cell growth via accumulation of DNA damage.

## Materials and Methods

### Cell culture

The osteosarcoma cell line 143B, Saos-2, and U2OS were purchased from the American Type Culture Collection (ATCC, Manassas, VA, USA). The HsOs1 cell line was purchased from the Riken cell bank (Tsukuba, Japan). Osteosarcoma cell lines were cultured in Dulbecco’s modified Eagle’s medium (DMEM) supplemented with 10% fetal bovine serum, penicillin (100 U/mL), and streptomycin (100 µg/mL). For analyzing DNA damage, recombinant Sonic Hedgehog protein (R&D Systems, Minneapolis, MN, USA), ATO (Nihon Shinyaku, Kyoto, Japan), and cisplatin (CDDP) (LKT Laboratories, Minneapolis, USA) were used. Cell lines were cultured in a humidified incubator with 5% CO_2_ at 37°C.

### Real-time polymerase chain reaction

Human osteosarcoma cells were cultured with or without 1 µM ATO. A vehicle (aqueous sodium hydroxide and hydrochloric acid to adjust to pH 7.5) was used as the control. Primer sets amplified amplicons of 150 to 200 bp in size. Polymerase chain reactions (PCRs) were performed using SYBR Green (BIO-RAD) on a MiniOpticon^TM^ machine (BIO-RAD). The comparative Ct (ΔΔCt) method was used to evaluate the fold change in mRNA expression using *β-actin* as the reference gene. All PCR reactions were performed in triplicate, with 3 different concentrations of cDNA. All primers were designed using Primer3 software (http://frodo.wi.mit.edu/cgi-bin/primer3/primer3.cgi). The following primers were used: *PTCH1*: 5′-TAACGCTGCAACAACTCAGG-3′, 5′-GAAGGCTGTGACATTGCTGA-3′; *GLI1*: 5′-GTGCAAGTCAAGCCAGAACA-3′, 5′-ATAGGGGCCTGACTGGAGAT-3′, *GLI2*: 5′-CGACACCAGGAAGGAAGGTA-3′, 5′-AGAACGGAGGTAGTGCTCCA-3′; *β-actin*: 5′-AGAAAATCTGGCACCACACC-3′, 5′-AGAGGCGTACAGGGATAGCA-3′.

Each experiment was performed in triplicate, and all experiments were performed 3 times.

### WST-1 assay

Human osteosarcoma cells were cultured with or without 1 µM or 3 µM ATO. An equivalent volume of vehicle (aqueous sodium hydroxide and hydrochloric acid to adjust to pH 7.5) was used as the control. The cells were treated with WST-1 substrate (Roche, Basel, Switzerland) for 4 h, washed with phosphate-buffered saline, and lysed to release formazan. Then, the cells were analyzed on a microplate reader (BIO-RAD, Hercules, CA, USA). Each experiment was performed in triplicate, and all experiments were performed 3 times.

### Colony formation assay

Cells were cultured in DMEM containing 0.33% soft agar and 5% fetal bovine serum, and plated on 0.5% soft agar layer. Cells were cultured in 6-well plates at a density of 5 × 10^3^ cells per well. Human osteosarcoma cells were cultured with or without 3 µM ATO. An equivalent volume of vehicle was used as the control. Fourteen days later, the number of colonies was evaluated. Each experiment was performed in triplicate, and all experiments were performed 3 times.

### Cell cycle analysis

Human osteosarcoma cells were cultured with or without 1 µM ATO. An equivalent volume of vehicle was used as the control. Cell cycle analysis was performed as previously reported [9]. Cells were collected, fixed with 70% ethanol for 2 h at 4°C, washed with phosphate-buffered saline, and treated with 500 µL staining buffer containing RNase A and 50 µg/mL propidium iodide (Wako Chemicals, Kanagawa, Japan). The DNA content was examined by flow cytometry using CyAn^TM^ ADP (Beckman Coulter, CA, USA) and Summit software (Beckman Coulter). Each experiment was performed in triplicate, and all experiments were performed 3 times.

### Comet assay

Human osteosarcoma cells were cultured with or without 3 µM ATO. An equivalent volume of vehicle was used as the control. Cells were trypsinized and electrophoresed on agarose gels as previously reported [26]. Tail moment (TM) and tail length (TL) were used to evaluate DNA damage in individual cells. Image analysis and quantification were performed using NIH ImageJ software. TM = % DNA in the tail × TL, where % of DNA in the tail = tail area (TA) × tail area intensity (TAI) × 100/(TA × TAI) + [head area (HA) × head area intensity (HAI)].

### Western blotting

Human osteosarcoma cells were cultured with or without 3 µM ATO. An equivalent volume of vehicle was used as the control. The cells were dissolved in NP40 buffer containing 0.5% NP40, 10 mM Tris-HCl (pH 7.4), 150 mM NaCl, 3 mM pAPMSF (Wako Chemicals, Kanagawa, Japan), 5 mg/mL aprotinin (Sigma, St. Louis, MO, USA), 2 mM sodium orthovanadate (Wako Chemicals), and 5 mM EDTA. Sodium dodecyl sulfate-polyacrylamide gel electrophoresis and immunoblotting were performed subsequently. The following antibodies were used: phospho-histone H2AX (Ser139) (γH2AX) (Cell Signaling Technology, MA, USA), cleaved caspase-3 (Asp175) (Cell Signaling Technology), poly (ADP-ribose) polymerase (PARP) (Cell Signaling Technology), Bcl-2 (Cell Signaling Technology), Bcl-xL (Cell Signaling Technology), SAPK/JNK (Cell Signaling Technology), Phospho-SAPK/JNK (Thr183/Tyr185) (Cell Signaling Technology), NF-κB p65 (Cell Signaling Technology), phospho-NF-κB p65 (Ser468) (Cell Signaling Technology), and tubulin (Santa Cruz, California, USA). Bands were visualized using the ECL chemiluminescence system (Amersham, Giles, UK).

### Xenograft model

143B cells (1 × 10^6^) and 100 µL Matrigel (BD, NJ, USA) suspension were subcutaneously inoculated into 5-week-old nude mice. The mice were randomly allocated to treatment with either ATO (10 µg/g) or an equivalent volume of vehicle (30 mM NaOH, pH 7.0). ATO and vehicle were administered intraperitoneally every day. ATO and vehicle treatment was started at 1 week after inoculation, at which time, the tumors had grown to a visible size. The tumor size was measured using the formula LW^2^ /2 (L and W represent the length and width of tumors, respectively). This study was carried out in strict accordance with the recommendations in the Guide for the Care and Use of Laboratory Animals of Kagoshima University. The animal experiment protocol was approved by the Institutional Animal Care and Use Committee, Graduate School of Medical and Dental Sciences, Kagoshima University (Permit Number: MD11017). All surgeries were performed under general anesthesia, and every effort was made to minimize the number of animals used and animal pain.

### Immunohistochemistry

ApopTag® Peroxidase In Situ Apoptosis Detection Kit was used for TUNEL staining according to the supplier’s protocol (MerckMillipore, Billerica, MA, USA). The sections were stained with methyl green (Merck-Chemicals, Darmstadt, Germany) to identify nuclei.

### Statistical analysis

All examinations were performed 3 times, except where otherwise stated, and all samples were analyzed in triplicate. All results are presented as mean (SD). Statistical differences between groups were assessed by Student’s *t*-test for unpaired data using Microsoft Office Excel (Microsoft, Albuquerque, NM, USA) and Kaplan 97.

## Results

### ATO prevents GLI transcription and proliferation of osteosarcoma cells

**Figure 1:**
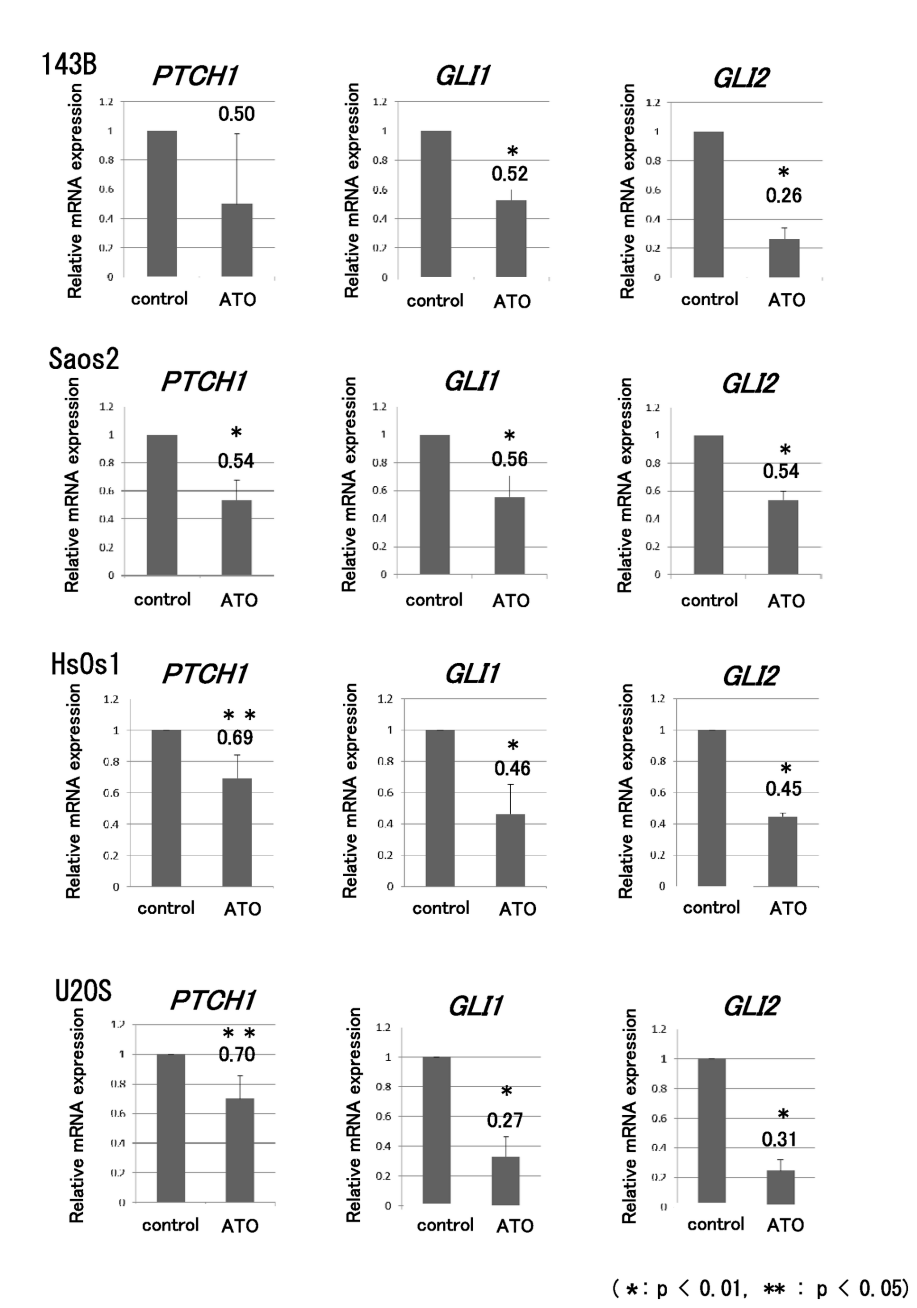
ATO prevents the transcription of GLI target genes. Human osteosarcoma cells were cultured with or without 1 µM ATO. An equivalent volume of vehicle was used as the control. Total RNA collected from osteosarcoma cell lines was examined by real-time polymerase chain reaction (PCR). A comparative Ct (ΔΔCt) analysis was performed to examine fold changes in mRNA expression compared with *β-actin*. Real-time PCR showed that ATO decreased the transcription of GLI target genes, including *PTCH1*, *GLI1*, and *GLI2*, in 143B, Saos2, HsOs1, and U2OS cells. The experiment was performed in triplicate with similar results (error bars represent mean [SD]) (*P < 0.01, **P < 0.05).

**Figure 2:**
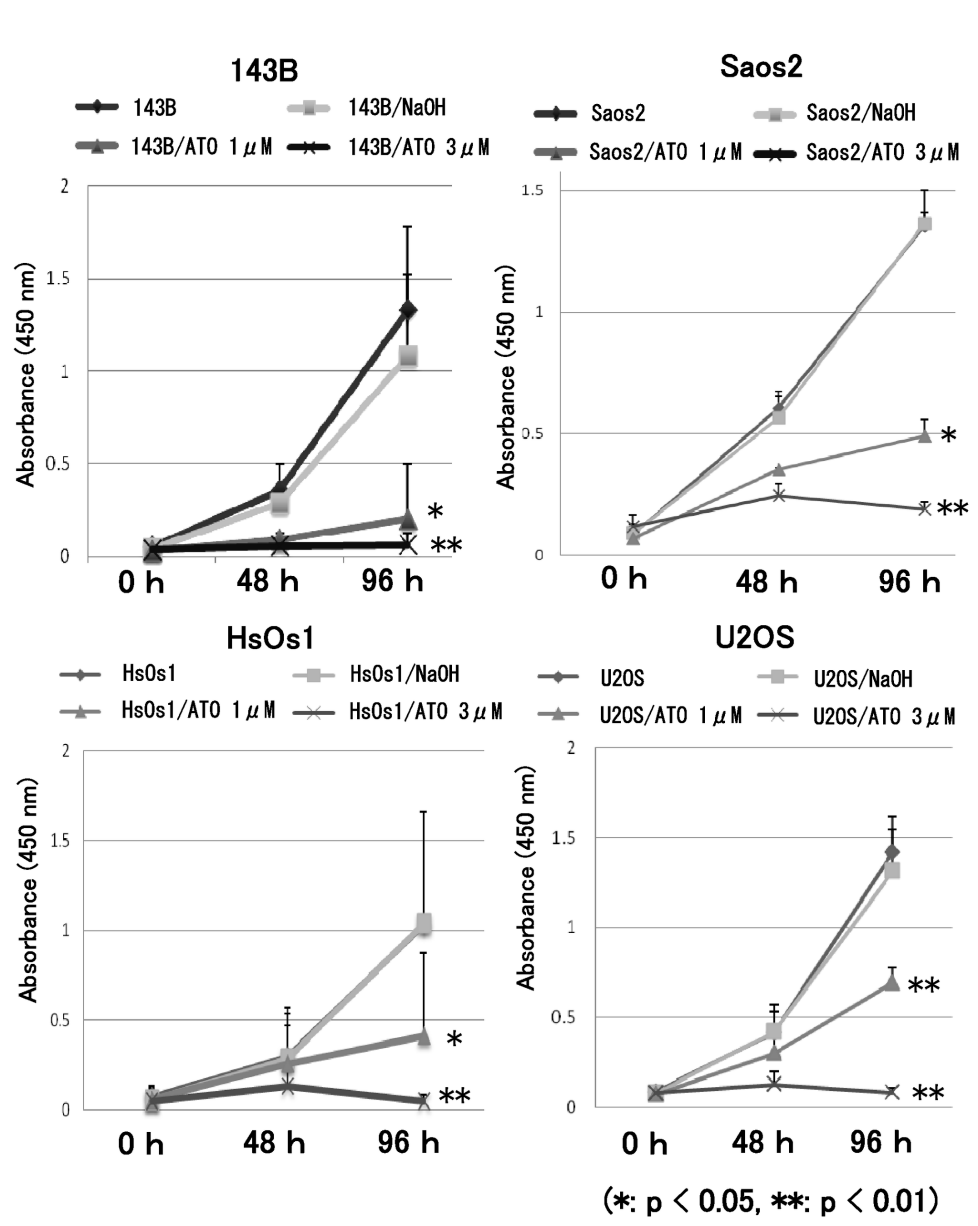
ATO prevents human osteosarcoma cell proliferation. WST assay showed that the growth of 143B, Saos-2, HsOs1, and U2OS cells was prevented by 1 µM or 3 µM ATO treatment for 96 h. An equivalent volume of vehicle was used as the control. The experiment was performed in triplicate with similar results (*P < 0.05, **P < 0.01) (error bars represent mean [SD]).

**Figure 3:**
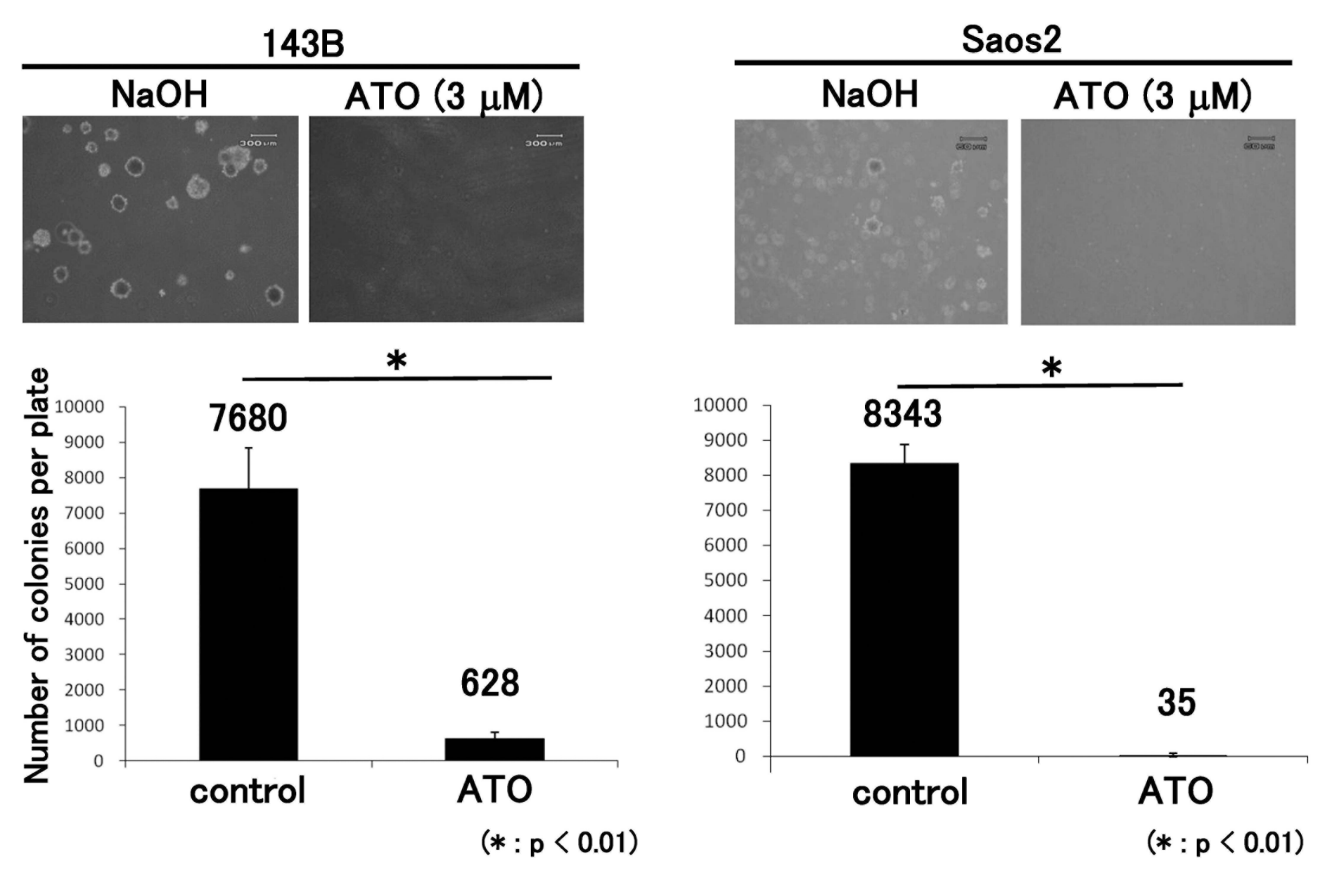
ATO inhibits anchorage-independent osteosarcoma growth. Treatment of 143B and Saos2 cells with 3 µM ATO reduced the number of colonies in soft agar at 14 days. An equivalent volume of vehicle was used as the control. These experiments were performed in triplicate with similar results (*P < 0.01) (error bars represent mean [SD]).

To determine whether ATO prevents GLI transcription in osteosarcoma cells, real-time PCR was performed for ATO-treated cells. Four human osteosarcoma cell lines showing upregulation of GLI transcription were examined [9,10]. The human osteosarcoma cell lines were treated with ATO at previously reported concentrations, which inhibit human cancer cell proliferation by inhibiting activation of the Hedgehog pathway [25]. Real-time PCR revealed that ATO prevented the transcription of GLI target genes, including *PTCH1*, *GLI1*, and *GLI2*, in human osteosarcoma cell lines (Figure 1). The WST-1 assay showed that proliferation of the 143B, Saos2, HsOs1, and U2OS cell lines was inhibited by ATO (Figure 2). We next evaluated the effects of ATO on anchorage-independent growth of osteosarcoma cells. The colony formation assay showed that ATO treatment decreased the number of colonies in soft agar (Figure 3). These findings showed that ATO treatment prevents GLI transcription and growth of osteosarcoma cells in vitro.

### ATO promotes DNA damage and apoptotic cell death

**Figure 4:**
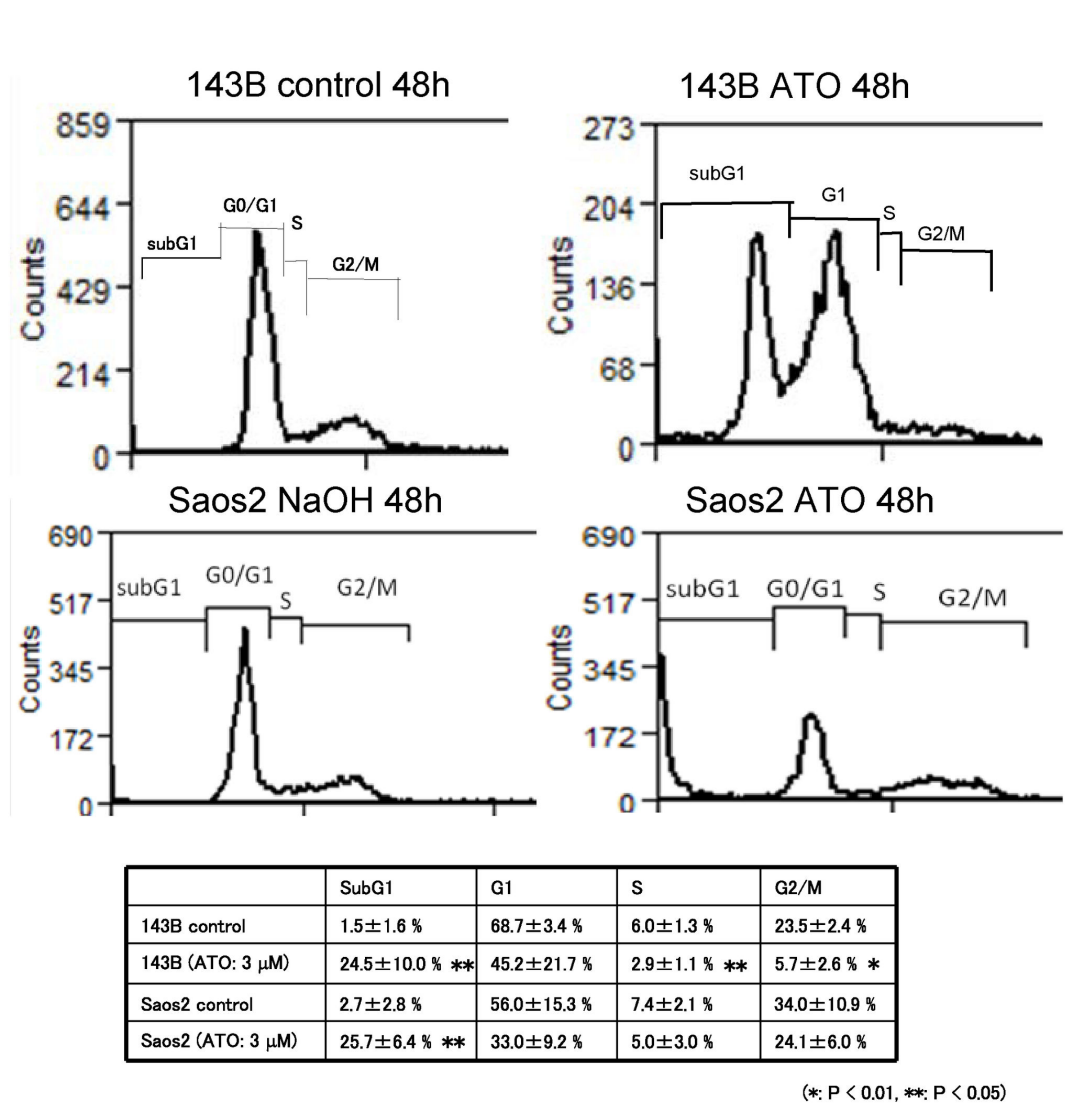
ATO promotes apoptotic cell death in human osteosarcoma cells. Human osteosarcoma cells were cultured with or without 1 µM ATO. An equivalent volume of vehicle was used as the control. Flow cytometric analysis was performed after ATO treatment for 48 h. ATO treatment significantly increased the Sub-G1 population of 143B and Saos2 cells. These experiments were performed in triplicate with similar results (*P < 0.01, **P < 0.05).

**Figure 5:**
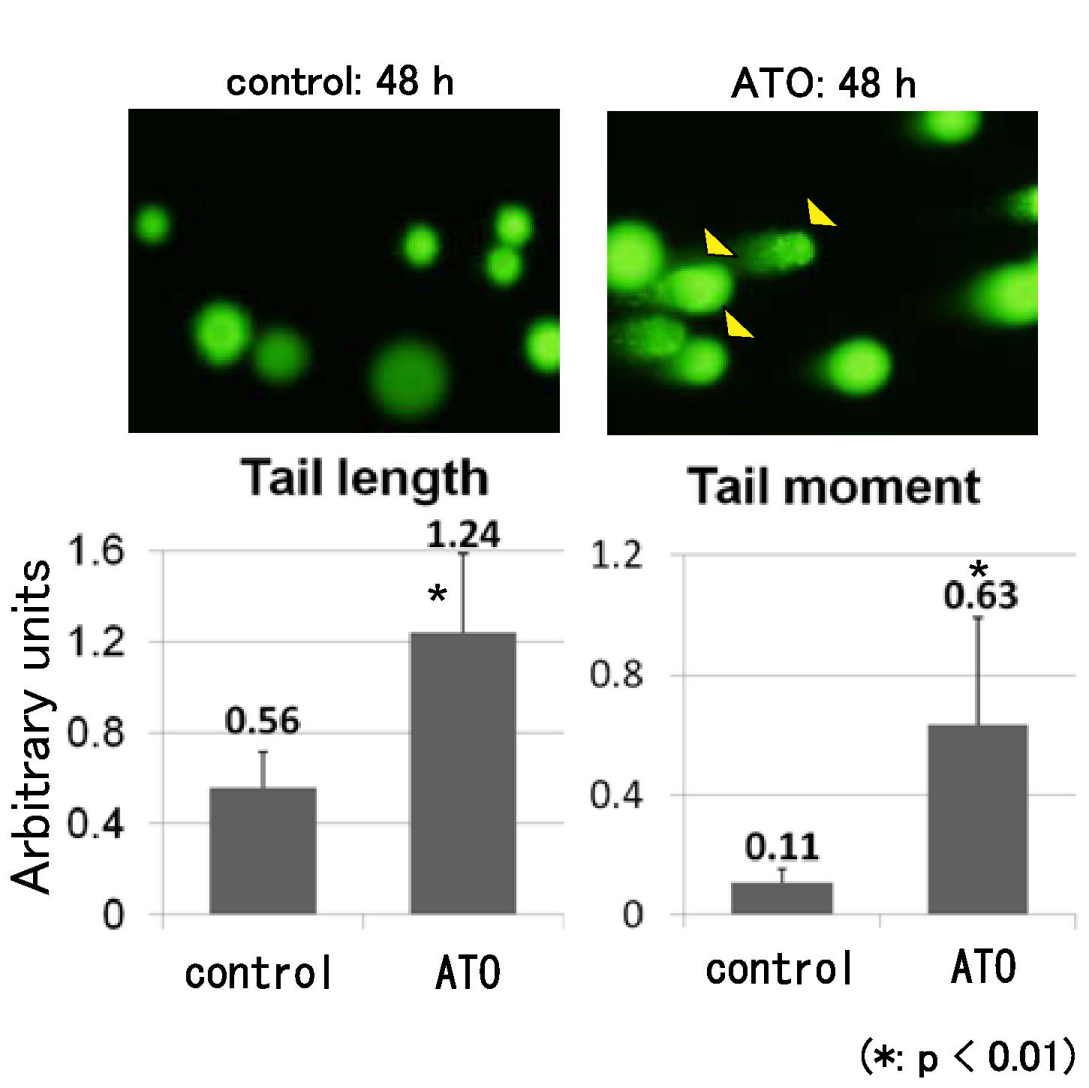
ATO elicits DNA damage in human osteosarcoma. COMET assay was performed to detect DNA damage in single cells after ATO treatment. 143B cells were treated with ATO (3 µM) or an equivalent volume of control vehicle for up to 48 h and analyzed by performing the COMET assay. Graphs represent DNA damage by tail length and tail moment, evaluated as described in the Materials and Methods section. These experiments were performed in triplicate with similar results (*P < 0.01).

**Figure 6:**
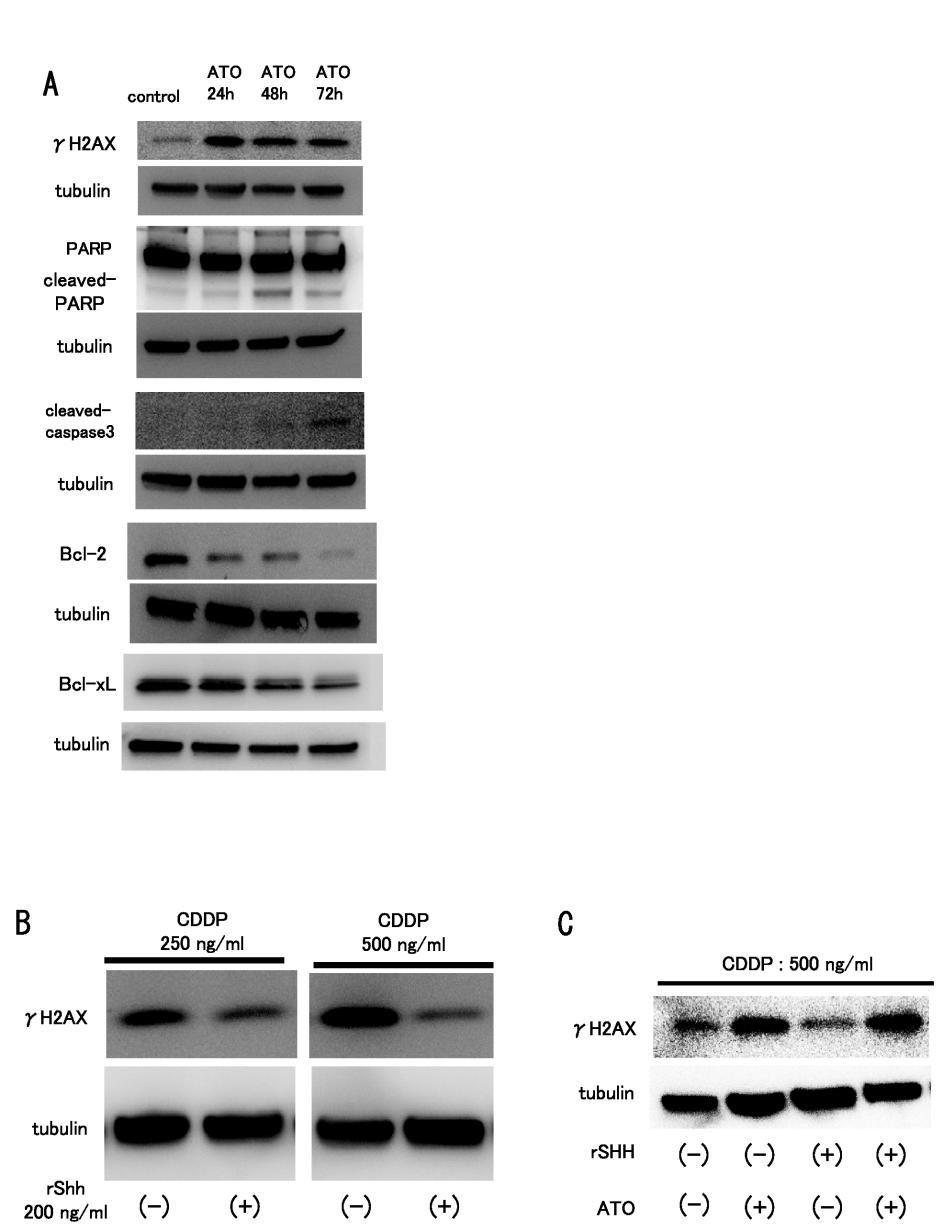
ATO elicits DNA damage and apoptosis. Human osteosarcoma cells were cultured with or without 3 µM ATO. An equivalent volume of vehicle was used as the control. Western blot analysis was performed 48 h and 72 h after ATO treatment. (A) Western blot analysis revealed that ATO treatment increased the protein levels of γH2AX, cleaved PARP, and cleaved caspase-3. ATO treatment decreased the protein levels of Bcl-2 and Bcl-xL. (B) Western blot analysis performed after cisplatin (CDDP) and recombinant human Sonic Hedgehog (rSHH) treatment showed that CDDP treatment upregulated the expression of γH2AX. Addition of Sonic Hedgehog decreased the expression level of γH2AX protein, which was upregulated by CDDP treatment. (C) Western blot analysis was performed following CDDP and recombinant human Sonic Hedgehog (rSHH) or ATO treatment. Addition of Sonic Hedgehog decreased the expression level of γH2AX protein, which was upregulated by CDDP treatment. Addition of ATO restored the γH2AX expression attenuated by rSHH treatment. These experiments were performed in triplicate with similar results.

To examine whether ATO treatment promoted cell death or cell cycle arrest, we performed flow cytometric analysis. The results showed that ATO treatment increased the population of sub-G1 cells (Figure 4). These findings show that ATO treatment promotes apoptotic cell death in osteosarcoma cells. To examine whether ATO promotes DNA damage, we performed a comet assay, which can be used to detect single cell DNA damage by the cellular elution pattern through agarose gels. The comet assay showed that ATO treatment altered the elution profiles (Figure 5). These findings show that ATO treatment promotes the accumulation of DNA damage in osteosarcoma cells. In addition, we used western blotting to examine the expression of DNA damage markers and apoptosis-related proteins after ATO treatment. Western blot analysis showed that ATO treatment increased the expression of γH2AX, a marker of double-strand breaks, cleaved poly (ADP-ribose) polymerase (PARP), and cleaved-caspase 3. In contrast, ATO treatment decreased the expression of Bcl-2 and Bcl-xL (Figure 6A). These findings suggest that ATO treatment promotes apoptotic cell death caused by accumulation of DNA damage.

It has been reported that ATO promotes apoptotic cell death and phosphorylation of JNK [27]. Although western blot analysis showed that ATO treatment increased the amount of phosphorylated JNK, inhibition of JNK activity had no effect on osteosarcoma cell proliferation with or without ATO, as seen with Ewing sarcoma cells (Figure S1) [23]. It has been reported that ATO treatment decreases the phosphorylation of NF-κB and promotes cell death [28]. Our findings showed that ATO treatment did not affect the status of NF-κB phosphorylation (Figure S1).

### Hedgehog signaling prevents DNA damage caused by CDDP treatment

To examine whether activation of Hedgehog signaling affects accumulation of DNA damage, we performed western blot analysis after cisplatin (CDDP) treatment. Western blotting showed that CDDP treatment upregulated the expression of γH2AX. Treatment with Sonic Hedgehog attenuated the upregulation of γH2AX (Figure 6B). In addition, we examined the effect of ATO treatment on the attenuation of DNA damage by Hedgehog activation. The attenuation of DNA damage caused by Hedgehog activation was reversed by ATO treatment (Figure 6C). These findings suggest that ATO promotes the accumulation of DNA damage by inhibiting Hedgehog signaling.

### ATO prevents osteosarcoma growth in vivo

**Figure 7:**
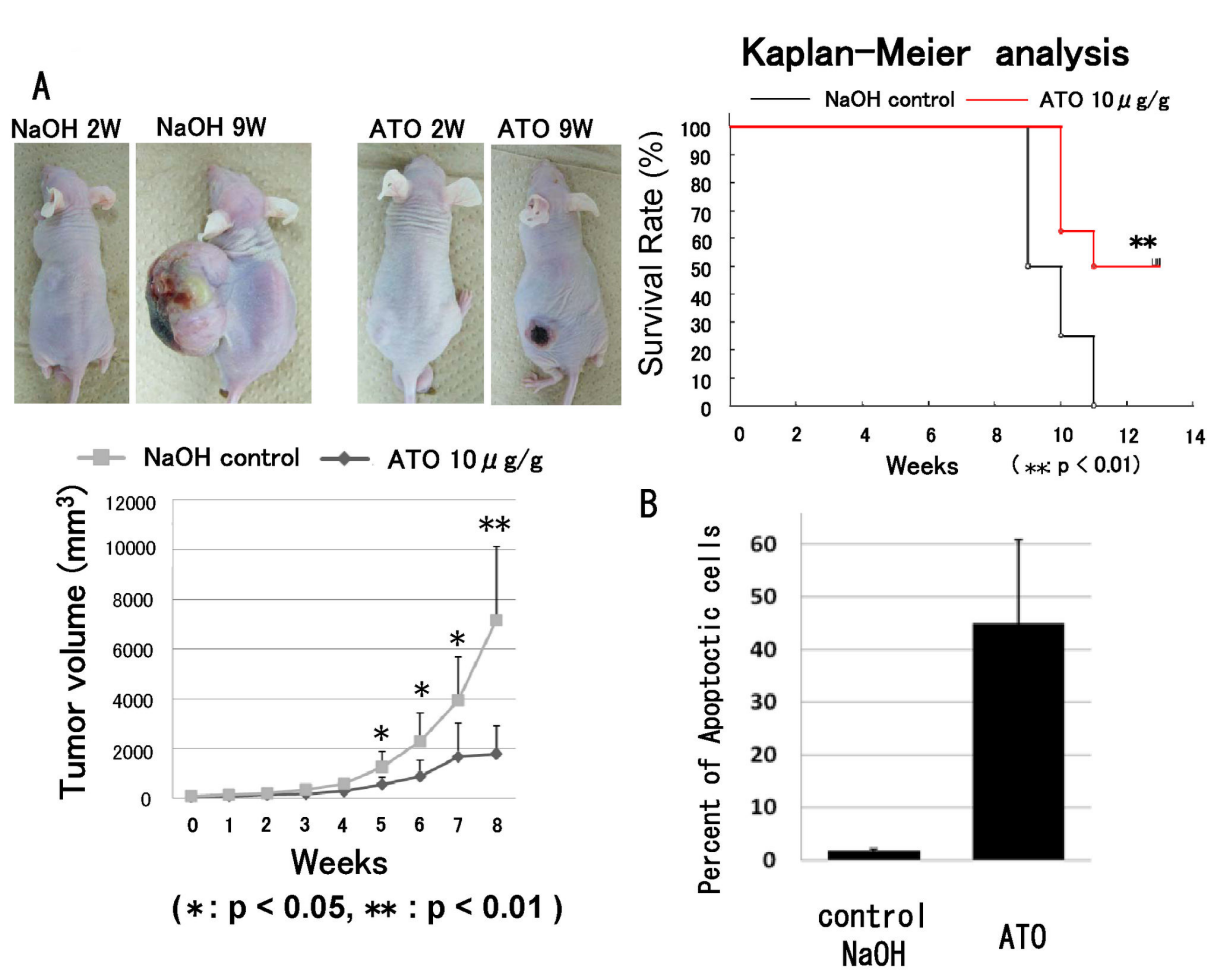
ATO prevents osteosarcoma growth in vivo. 143B cells (1 × 10^6^) were subcutaneously inoculated into nude mice. Tumor volume was calculated weekly using the formula LW^2^ /2 (where L and W represent the length and width of tumors). Seven days after inoculation, the tumor volume was set as 1 and was evaluated at different time points. (A) ATO treatment inhibited tumor growth as compared with control (*P < 0.05 or **P < 0.01) (error bars represent mean [SD]). Kaplan-Meier analysis revealed that ATO treatment provided a significant survival benefit (**P < 0.01). (B) Apoptotic cell death in the tumors was analyzed by TUNEL staining, which showed that ATO treatment increased apoptotic cell death in vivo (*P < 0.05 or **P < 0.05) (error bar indicates SD).

143B osteosarcoma cells were intradermally inoculated into nude mice, and palpable tumors were formed within 7 days. Then, ATO or an equivalent volume of vehicle was injected intraperitoneally. The injections were administered every day. Compared with vehicle treatment, treatment with ATO significantly prevented tumor growth (Figure. 7). Kaplan-Meier analysis showed that ATO treatment provided a significant survival benefit (Figure 7A). TUNEL staining showed that ATO treatment induced apoptotic cell death. The number of apoptotic cells was significantly increased in ATO-treated tumors (Figure 7B).

## Discussion

We and other researchers have previously reported that inhibition of the Hedgehog pathway prevented the growth of osteosarcoma cells [9,10,29]. In particular, we showed that knockdown of GLI2 prevented osteosarcoma cell growth in vitro and in vivo [9]. ATO prevents Ewing sarcoma, medulloblastoma, and basal cell carcinoma growth by inhibition of GLI transcription [23–25]. To apply our previous findings in clinical settings, we examined the effects of ATO in human osteosarcoma. We showed that ATO prevents the transcription of GLI target genes and promotes apoptotic cell death in osteosarcoma cells as a result of accumulation of DNA damage. In addition, ATO re-induces the accumulation of DNA damage attenuated by recombinant Sonic Hedgehog treatment. These findings suggest that ATO inhibits the activation of Hedgehog signaling and promotes apoptotic cell death in osteosarcoma cells as a result of accumulation of DNA damage. In addition, our findings showed that ATO decreased the expression of Bcl-2 and Bcl-xL. GLI1 and GLI2 upregulate the transcription of Bcl-2 and Bcl-xL [30–33]. Inhibition of the Hedgehog pathway by ATO treatment may downregulate Bcl-2 and Bcl-xL to promote apoptotic cell death in osteosarcoma cells. Singh et al. reported that ABCG2, a drug transporter protein, is a direct transcriptional target of Hedgehog signaling [33]. These findings suggest that activation of Hedgehog signaling promoted the export of CDDP by the ABCG2 transporter and reduced the accumulation of DNA damage in osteosarcoma cells. Inhibition of the Hedgehog pathway by ATO treatment may be useful as an adjunct treatment to conventional chemotherapy for osteosarcoma. In addition, several molecular mechanisms have been reported for inhibition of the Hedgehog pathway by ATO. Kim et al. reported that ATO prevented growth of medulloblastoma by reducing stability of GLI2 protein and ciliary accumulation of GLI2 [25]. Elspeth et al. reported that ATO prevents growth of cancer cell lines and Ewing sarcoma by inhibiting GLI transcription through direct binding to GLI [23]. Although there were some discrepancies related to the mechanism of Hedgehog pathway inhibition by ATO, these studies independently suggest that ATO inhibits malignant tumor growth by inhibition of the Hedgehog pathway at the level of GLI transcription factors. These mechanisms may prevent osteosarcoma growth after ATO treatment. Because aberrant activation of the Hedgehog pathway has been implicated in several malignant tumors, the pharmaceutical industry has invested in the development of Hedgehog pathway inhibitors. SMO inhibitors have been evaluated in recent clinical trials [34,35]. However, treatment with SMO inhibitors showed a lack of efficacy in a portion of patients. Investigation of the underlying mechanism revealed that the patient tumors showed a mutation in SMO that prevented binding of the SMO inhibitors to SMO [15]. Several genes with potential mutations within SMO and downstream of SMO have been found [16–21,36]. In addition, non-Hedgehog pathway-mediated activation of GLI transcription has been reported [37–41]. In this regard, direct GLI inhibition by ATO is likely to be useful for treating tumors with mutations within or downstream of SMO. For example, inhibition of GLI, but not SMO, inhibited tumor growth in myeloid leukemia, colon carcinoma, hepatocellular carcinoma, and osteosarcoma [9,42–44]. Originally, arsenic was used in the 17^th^ century to treat leukemia. ATO has been approved for the treatment of intractable acute promyelocytic leukemia in Japan. Our findings suggest that ATO is one of the most suitable molecular target reagents for inhibiting the Hedgehog pathway in human osteosarcoma. We have now obtained approval from the ethics committee for clinical research, Kagoshima University, to use ATO for treating patients with intractable osteosarcoma.

We examined whether the inhibitory effect of ATO on osteosarcoma growth is mediated, at least in part, by JNK or NF-κB [45–47]. As previously reported, treatment with ATO increased JNK phosphorylation. However, treatment with a JNK inhibitor did not prevent osteosarcoma growth. In contrast, treatment with ATO did not affect NF-κB activation. These findings indicate that JNK or NF-κB activation does not affect the cytotoxicity of ATO in human osteosarcoma.

For in vivo examinations, we administered ATO intraperitoneally at 10 mg/kg body weight, as previously reported [25]. Kim et al. examined the ATO levels in mouse sera collected after ATO administration by injection at 10 mg/kg body weight. The peak concentration following intraperitoneal injection at 10 mg/kg was 2.6-fold higher than the peak plasma levels in human patients following intravenous ATO injection at a dose of 0.15 mg/kg body weight [48]. Area under the curve calculations revealed that the total exposure to ATO in mice at the 10 mg/kg dose was 2-fold higher than that in patients. To decrease the ATO concentration, combinations of drugs that inhibit other Hedgehog signaling components, including SMO inhibitors, were used to achieve greater pathway inhibition at lower ATO concentrations [25]. In addition, Kim et al. reported that combined use of ATO and itraconazole, a commonly used antifungal that inhibits SMO by a mechanism distinct from that of cyclopamine and other known SMO antagonists, decreases the dose of ATO and itraconazol required to prevent medulloblastoma and basal cell carcinoma growth associated with acquired resistance to SMO antagonists [24].

In summary, our findings showed that ATO inhibits the Hedgehog pathway and human osteosarcoma cell growth in vitro and in vivo. The combined administration of conventional anticancer agents or other Hedgehog pathway inhibitors with ATO may be valuable for treating osteosarcoma patients.

## Supporting Information

Figure S1 Western blot analysis showed that ATO treatment decreased the expression of phosphorylated JNK. Western blot analysis showed that ATO treatment did not affect the expression levels of NFκB and phosphorylated NFκB proteins. WST assay showed that JNK inhibitor did not affect the proliferation of osteosarcoma cells.
(TIF)
