# Supplementary figures and images for: Arsenic Trioxide Prevents Osteosarcoma Growth by Inhibition of GLI Transcription via DNA Damage Accumulation
Source: PLoS One. 2013 Jul 8;8(7):e69466. doi: 10.1371/journal.pone.0069466 (PMC3704531; doi:10.1371/journal.pone.0069466)

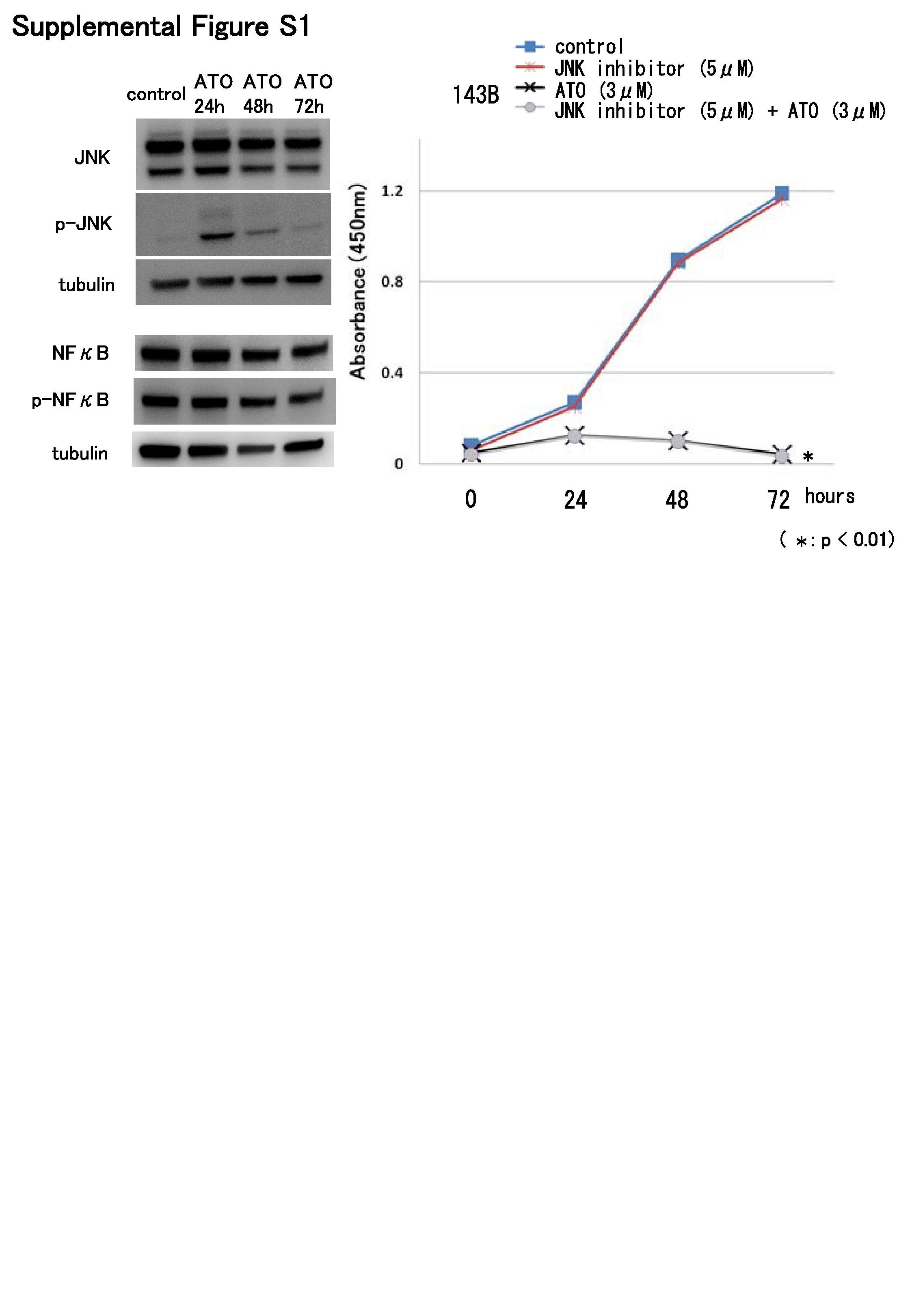

Supplement: Figure S1 — Western blot analysis showed that ATO treatment did not affect the expression levels of NFκB and phosphorylated NFκB proteins. WST assay showed that JNK inhibitor did not affect the proliferation of osteosarcoma cells. (TIF) [file pone.0069466.s001.tif]
